# Theory of Semiflexible Filaments and Networks

**DOI:** 10.3390/polym9020052

**Published:** 2017-02-05

**Authors:** Fanlong Meng, Eugene M. Terentjev

**Affiliations:** 1 Cavendish Laboratory, University of Cambridge, Cambridge CB3 0HE, UK; fanlong.meng@physics.ox.ac.uk; 2 Rudolf Peierls Centre for Theoretical Physics, University of Oxford, Oxford OX1 3NP, UK

**Keywords:** theory, semiflexible chain, semiflexible filament network, transient network

## Abstract

We briefly review the recent developments in the theory of individual semiflexible filaments, and of a crosslinked network of such filaments, both permanent and transient. Starting from the free energy of an individual semiflexible chain, models on its force-extension relation and other mechanical properties such as Euler buckling are discussed. For a permanently crosslinked network of filaments, theories on how the network responds to deformation are provided, with a focus on continuum approaches. Characteristic features of filament networks, such as nonlinear stress-strain relation, negative normal stress, tensegrity, and marginal stability are discussed. In the new area of transient filament network, where the crosslinks can be dynamically broken and re-formed, we show some recent attempts for understanding the dynamics of the crosslinks, and the related rheological properties, such as stress relaxation, yield stress and plasticity.

## 1. Introduction

What is today routinely called “semiflexible filaments”, 30 years ago was more frequently referred to as polymer chains with high bending modulus. The classical theory of polymers starts from the ideal chain models (such as beads and springes, or freely-jointed rods) and the Edwards Hamiltonian [1] that accounts for the connectivity of monomers:

(1) $$H=\frac{3k_{B}T}{2b^{2}}\sum_{j=1}^{N}{\left(r_{j}-r_{j-1}\right)}^{2}=\frac{3k_{B}T}{2b}\int_{0}^{L}ds{\left|\frac{dr(s)}{ds}\right|}^{2},$$

where *b* is the Kuhn length (essentially the monomer size) and *N* the number of monomers, each positioned at r(s) along the chain. The contour length of this chain is L=Nb. This Gaussian Hamiltonian gives the average end to end distance of the chain $\langle {\left[r\left(N\right)-r\left(0\right)\right]}^{2}\rangle =b^{2}N$ in full analogy with free diffusion laws. It is also the basis for the dynamic theories such as Rouse model and its versions. However, very soon people realized that adding the bending rigidity to this ideal chain would not only lead to many interesting theoretical results and analogies, but also would better describe many actual polymer chains and filaments. We review this in detail in the next section.

As with ordinary, fully flexible polymer chains, the main avenue of further research went towards the dense systems, when the chains begin to interact with each other. The interactions range from the plain excluded volume for two monomers approaching contact, starting with a pair-interaction term $∼\delta [r\left(s\right)-r\left(s^{\prime }\right)]$ added to the Edwards Hamiltonian, to various long-range interactions leading to additional physics and phase transformations. In the case of chains with bending rigidity, at higher density one often encounters the nematic liquid crystal phases [2,3,4,5], where chains fold into tight hairpins with long straight segments aligned [6,7,8]. For very stiff chains, when backfolding (hairpins) is not allowed, the analogy with vortex lines in superconductors has led to another successful stream of theoretical research using the transverse chain excursions away from the mean centerline $r_{⊥}\left(s\right)$ as the field variable [9,10]. The issues of semiflexible chains confined in limited and shaped volume were recently reviewed [11].

Here we will not touch these very interesting topics. Our aim is to examine the properties of an isolated filament (subject to various external forces and extensions), and then proceed to the crosslinked networks of such filaments, in all cases remaining in the regime of low total volume fraction. This is closely corresponding to many biological scaffolding systems, which underpins the current active interest in this class of materials.

## 2. A Single Semiflexible Filament

Semiflexible filaments can be categorized into natural, mainly biological ones such as DNA, actin, collagen, microtubules, intermediate filaments, etc., and the synthetic filaments such as carbon nanotubes or stiff polymer chains. The main physical feature of this class of polymer chains is that there is a finite bending rigidity, such that their mechanical properties are determined by the competition between the elastic potential energy of bending and the entropic free energy arising from thermal fluctuations of their conformations. There is a possibility to have elastic energy of extension added to a model, but our first aim is to examine filaments that are inextensible.

**Figure 1:**
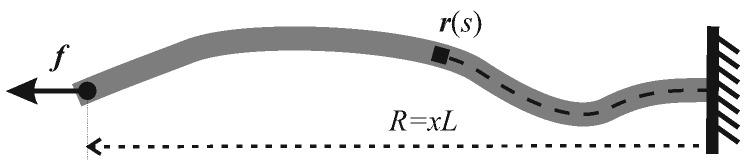
Sketch of a semiflexible filament under a tensile force *f*, illustrating the notations used in the wormlike chain model and the analysis below.

The wormlike chain (WLC) model, proposed by Kratky and Porod in 1949 [12], is widely used for describing the elasticity of a single semiflexible filament, since the work of Fixman and Kovac in 1973 [13]. As sketched in Figure 1, a filament with its contour length as *L* is coordinated by r(s), where 0≤s≤L is the arc-length coordinate along the chain. By assuming a finite bending modulus of the filament, its Hamiltonian can be expressed as

(2) $$\begin{array}{ccc} H[r(s)] & = & \frac{\kappa }{2}\int_{0}^{L}ds{\left(\frac{d^{2}r}{ds^{2}}\right)}^{2}, \end{array}$$

where $d^{2}r/ds^{2}$ denotes for local curvature of a curve, and *κ* is the bending modulus. In classical elasticity, the bending modulus of a filament is given by the Young modulus of its material and the shape of its cross-section: κ=YI, where the second moment of filament cross-section $I=\int y^{2}dxdy$; for an elastic tube of outer radius *a* and inner radius *b* (the wall thickness a−b) the bending modulus will be $\kappa =\left(\pi /4\right)Y\left(a^{4}-b^{4}\right)$. Persistence length, defined as $l_{p}=\kappa /k_{B}T$, is a key quantity for describing whether a chain is regarded as flexible ($L\gg l_{p}$), semiflexible ($L∼l_{p}$), or rigid ($L\ll l_{p}$). As the persistence length is a function of the temperature, a chain can become more flexible under high temperature, and vice versa. As noticed from how the conformation is parameterized in WLC model by the coordinate r(s), the semiflexible chain is naturally assumed as locally inextensible, i.e., ${\left(dr/ds\right)}^{2}=1$, for all *s*.

**Figure 2:**
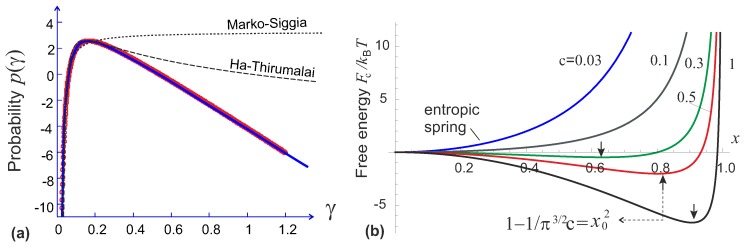
(**a**) The result of numerical calculation of the integral Equation (6) plotted as a universal function of parameter *γ*. The solid line drawn through these points is the analytical expression Equation (7); (**b**) Plots of the filament free energy, Equation (8), for several vaues of stiffness parameter *c* labelled on the plot. The arrows point at the force-free filament extension $x_{0}\left(c\right)$ that occur for stiffer filaments with $c>\pi^{-3/2}$.

As mentioned at the beginning, except for the bending energy defined above, the other important energy source of a semiflexible chain is thermal fluctuation of its conformations, or simply the conformational entropy. This can be more clearly understood by formulating the free energy of a semiflexible chain, F(R), as a function of its end-to-end separation, R=r(L)−r(0), which is useful in analyzing its mechanic properties, such as the force-extension relation. Alternatively, it can be re-expressed as a function of the non-dimensional ’end-to-end factor’ x=R/L. The free energy of a semiflexible chain is

(3) $$\begin{array}{c} F=-k_{B}T\ln p\left(x\right), \end{array}$$

where the probability p(x) is the partition function at a constrained end-to-end distance, expressed by the standard ’Edwards path integral’:

(4) $$\begin{array}{c} p\left(x\right)\propto \int Dr\left(s\right)\;e^{-H[r]/k_{B}T}\delta \left[{\left(\frac{dr}{ds}\right)}^{2}-1\right],\;\;\mathrm{with}\;\;\;x=\frac{1}{L}\int_{0}^{L}ds\left(\frac{dr}{ds}\right), \end{array}$$

where the local inextensibility is incorporated as a delta-function constraint in the path integral. However, it is difficult to obtain an analytic free energy expression of an inextensible chain defined above: although the bending Hamiltonian is quadratic in the field r(s), the inextensibility constraint makes this classical problem highly non-linear. One of the ways for resolving this difficulty is by using the concept of mean extensibility (often called the ’mean field approximation’ in this context), where the total length of the chain *L* is conserved, while local fluctuations are allowed. Although mathematically this is an approximation – physically this limit is perhaps a more reasonable model of a semiflexible chain or filament. The idea of mean extensibility is simple: the Fourier transformation of *δ*-function in Equation (4) is

(5) $$\begin{array}{ccc} \delta \left[{\left(\frac{dr}{ds}\right)}^{2}-1\right] & = & \int D\phi \left(s\right)e^{i\phi \left(s\right)\left[{\left(dr/ds\right)}^{2}-1\right]}\;, \end{array}$$

where ϕ(s) is introduced as an auxiliary field, imposing the original constraint at each point *s* along the filament. If we replace ϕ(s) with a constant value *ϕ* (the ’mean field’), then the chain can be extensible locally, but with the average inextensibility will be maintained: $\langle {\left(dr/ds\right)}^{2}\rangle =1$. The validity of this approximation can be checked by calculating the fluctuations of the local length. As discussed in Ref. [14], if a chain is divided into *N* segments with equal length as L/N, then the fluctuation in the local arc-length of an individual segment, Δ, can be expressed as ${\langle \Delta^{2}\rangle }^{1/2}=L{\left[\left(N-1\right)\left(N+1\right)\right]}^{1/2}/N$, which is equal to L/N for N→∞; the fluctuations are well-bounded. With this approximation, the probability function (4) can be reduced to a compact integral in infinite limits:

(6) $$\begin{array}{c} p\left(\gamma \right)=\int d\phi e^{i\gamma \phi }{\left(\frac{\sqrt{i\phi }}{\sin\sqrt{i\phi }}\right)}^{3/2}, \end{array}$$

where the single non-dimensional parameter $\gamma =\kappa \left(1-x^{2}\right)/2k_{B}TL=\left(l_{p}/2L\right)\left(1-x^{2}\right)$ measures the deviation of the chain from its full extension, and at the same time – encodes the effective stiffness of the filament [14]. It turns out that a simple analytical expression

(7) $$p\left(\gamma \right)=\exp\left[-\pi^{2}\gamma -\frac{1}{\pi \gamma }\right]$$

captures the full range of Equation (6). A numerical calculation of the probability function is shown in Figure 2a. This numerical integration is quite non-trivial; it uses the fact that the imaginary part of the integrand in Equation (6) is antisymmetric in *ϕ*, while the (symmetric) real part has several points on the *ϕ*-axis where an artefact of discontinuous sign-reversal occurs in numerical evaluation. So the integral has to be evaluated piece-wise, correcting for these artefacts. The analytical interpolation Equation (7) is also plotted in Figure 2a, shown to follow the numerical result with high precision. The close match across the whole range of *γ* suggests that this is, in fact, an exact analytical result of integration (which we simply didn’t know how to calculate). This allows an immediate evaluation of the free energy of semiflexible filament $F_{c}\left(x\right)$:

(8) $$F_{c}\left(x\right)=k_{B}T\pi^{2}c\left(1-x^{2}\right)+\frac{k_{B}T}{\pi c(1-x^{2})},$$

where the non-dimensional stiffness parameter $c=l_{p}/2L$ is the single factor that determines the properties of an inextensible filament. Note that this expression has no approximations (apart from the ’mean-field’ approximation for an auxiliary field *ϕ*), and so it is equally valid in the limit of flexible chains (c≪1,x≪1), giving the classical entropic-spring result, in the limit of highly stretched flexible chain (c≪1,x→1), and also in the limit of rigid athermal elastic rod (c≫1,x→1).

### 2.1. Models of the Force-Extension Relation

**Figure 3:**
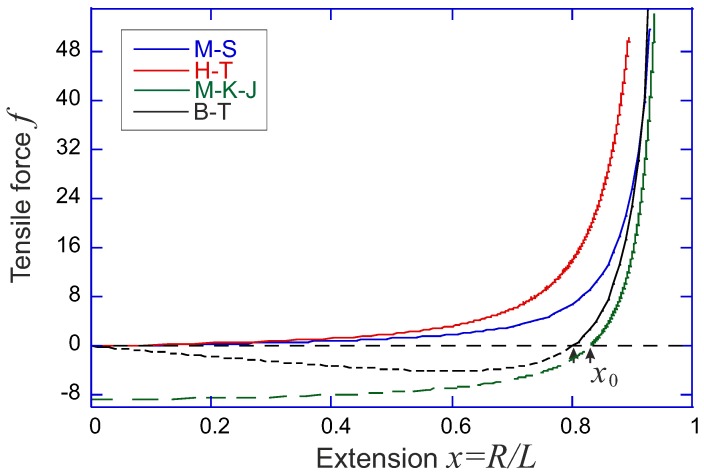
Comparison of tensile force *f* (plotted in units of $k_{B}T/l_{p}$) against the filament extension factor x=R/L, for the Marko-Siggia, Ha-Thirumalai, MacKintoish-Käs-Janmey and Blundell-Terentjev models. In all cases the filament stiffness is such that $L=l_{p}$ (although the M-S model does not depend on this ratio). Dashed lines of the M-K-J and B-T models show the outward force in the compression regime with $x<x_{0}$ (the effect of zero force at x=0, in the full closed loop configuration of an elastica of the B-T model is because the force in Equation (12) is defined along the tangent of the first/last monomer); the H-T model does not predict a zero-force average filament extension, and therefore has no compression regime.

Over the years, several famous analytical forms of the force-extension relation of a semiflexible chain as a function of its end-to-end distance have been developed (comparison shown in Figure 3). We discuss these force-extension expressions here, while the corresponding free energy in each case could be obtained by an integral of the force over the extension. In particular, when we compare with the numerical result of Equation (6) in Figure 2a, the constrained free energy expressions are obtained by integration, and then raised to exponent to get a non-normalized probability plotted.

*Marko-Siggia model* [10]. In 1995, Marko and Siggia proposed an approximate force-expansion relation as:

(9) $$\begin{array}{c} f\left(x\right)=\frac{k_{B}T}{l_{p}}\left(x+\frac{1}{4{\left(1-x\right)}^{2}}-\frac{1}{4}\right)\;. \end{array}$$

It originates from the high-extension limit of the WLC model, which was interpolated such that it also matches the low-force limit of entropic spring. This expression works very well for chains near full extension, and has a long and successful history of application in many experimental situations [15]. For instance, most values of the persistence length of various biological filaments in the literature are obtained by fitting force-extension curves with the Marko-Siggia expression (9). When we examine this expression, it turns out that it is valid for a long, flexible chain in the highly stretched limit; in other words, at γ→0 in Equations (7) and (8).

*Ha-Thirumalai model* [16]. In 1997, Ha and Thirumalai proposed another approximate force-expansion relation by numerically solving:

(10) $$\begin{array}{ccc} f(x) & = & 2\lambda x,\;\;\;\;\mathrm{where}\;\lambda \left(f\right)\;\mathrm{satisfies}:\;\;\;\;\frac{f^{2}}{4\lambda^{2}}+\frac{3}{4}\sqrt{\frac{2k_{B}T}{l_{p}\lambda }}\coth\left(\frac{\sqrt{\lambda }L}{\sqrt{2k_{B}Tl_{p}}}\right)\;=\;1. \end{array}$$

This expression, as well as the Marko-Siggia model, and also the Odijk model [17], all predict the limiting behavior of $f\to \left(k_{B}T/l_{p}\right){\left[1-x\right]}^{-2}$ at x→1, with a slightly different numerical factor each. This is an inherent feature of the inextensible WLC model in the limit of high extension when the transverse excursions of the chain are small (certainly no ’overhangs’ are possible in the chain configuration). However, slightly different values of $l_{p}$ would result by equally successfully fitting the force-extension curves of chains and filaments by these expressions. In terminology of Equations (7) and (8), these limits correspond to the regime γ≪1, that is: a flexible chain at high extension. The validity of Ha-Thirumalai model is expanded to a wider range of *γ* compared with that by Marko and Siggia, as shown in Figure 2a.

*MacKintosh-Käs-Janmey model* [18]. Another way of evaluating the equilibrium elastic response of filaments to tensile force *f*, starting from the same WLC model, is by applying equipartition to each bending mode in Fourier space, which is nicely reviewed in [19] by Broedersz and Mackintosh, and in [20] by Palmer and Boyce. Again, using the mean-field constraint on chain extensibility, but a different way of performing the summation of Fourier modes, the end-to-end factor is expressed as a function of applied force:

(11) $$\begin{array}{c} x=1-\frac{L}{l_{p}\pi^{2}}\sum_{n}\frac{1}{n^{2}+\xi^{2}}=1-\frac{L}{l_{p}\pi^{2}}\left(\frac{\pi \xi \coth(\pi \xi )-1}{2\xi^{2}}\right), \end{array}$$

where the shorthand $\xi =\left(L/\pi \right)\sqrt{f/k_{B}Tl_{p}}$. The force-extension relation can be obtained by numerically inverting the relation between *x* and *f* (Palmer and Boyce [20] offer a rather accurate analytical interpolation using Padè approximation, valid in the tensile deformation regime). In the limit of high extensions, the divergent tensile force follows the same characteristic limit as all other models: $f\to \left(k_{B}T/4l_{p}\right){\left[1-x\right]}^{-2}$. A very important factor, missing in earlier models, is the natural length of the filament: the force-free average end-to-end extension we labelled as $x_{0}$ in Figure 2b. Palmer and Boyce give it as $x_{0}=1-L/6l_{p}$ for this model (e.g., at $L=l_{p}$ we have $x_{0}\approx 0.83$). In the linear regime (at small deformations around $x_{0}$) the filament responds with the spring constant 90 $k_{B}Tl_{p}^{2}/L^{4}$.

*Blundell-Terentjev model* [14]. Equations (7) and (8) constitute the model of Blundell and Terentjev, which has an advantage of being fully analytical and capturing the right physics in both the low and the high temperature limits, as well as in tension and compression/buckling regimes up to the highly bent elastica limit [21]. The force-extension relation is written as

(12) $$\begin{array}{c} f\left(x\right)=-\frac{\pi^{2}k_{B}Tl_{p}}{L^{2}}x+\frac{4k_{B}T}{\pi l_{p}}\frac{x}{{\left(1-x^{2}\right)}^{2}}, \end{array}$$

where an internal elastic energy of a bent filament is obtained in the limit T→0 for a fixed bending modulus $\kappa =k_{B}Tl_{p}$. As always for theories based on WLC, the finite extension limit gives the divergent force scaling: $f\to \left(k_{B}T/\pi l_{p}\right){\left[1-x\right]}^{-2}$. For a very short or stiff filament ($L\ll l_{p}$), the internal potential dominates over the entropic effects, and the filament can be treated as a rigid athermal rod. The zero-force average natural extension is $x_{0}^{2}=1-2L/\pi^{3/2}l_{p}$ (e.g., at $L=l_{p}$ we have $x_{0}\approx 0.80$). As shown in Figure 2b, if the ratio $c=l_{p}/2L$ is below the critical value of $\pi^{-3/2}$, the minimum of the free energy will be located at x=0, i.e., the filament can be regarded as a flexible chain and reproduce a Gaussian limit upon first-order expansion (with the entropic spring constant $4k_{B}T/\pi l_{p}L$).

### 2.2. Mechanical Response of a Semiflexible Filament

The characteristic response of a chain or filament to a tensile force has been widely explored in the literature and remains the basis of determining the persistence length $l_{p}$, usually by fitting the M-S model. For the filaments that are stiff enough to have a non-zero average natural extension $x_{0}$, the response to a compression force also becomes relevant. When a rigid elastic rod is compressed, it will buckle if the compression force exceeds what is known as the Euler buckling threshold force $f_{c}=\pi^{2}\kappa /L^{2}$ [22]. Similar response also appears in the case of a semiflexible filament, although in this case the thermal fluctuations result in a finite range of buckling force, and the associated hysteresis. The free energy $F_{c}\left(x\right)$ given in Equation (8) has the minimum located at $x_{0}$. The value of the free energy at x=0 decreases with the increase of the compression force until it becomes a metastable state; in other words, the Euler buckling of a semiflexible filament is a typical first order phase transition [19,21]. The equilibrium threshold force for buckling is obtained as

(13) $$\begin{array}{c} f_{c}^{*}=\frac{\pi^{2}k_{B}Tl_{p}}{L^{2}}\left[1-\alpha {\left(\frac{L}{l_{p}}\right)}^{\nu }\right], \end{array}$$

where the bending modulus *κ* is replaced by the persistence length $k_{B}Tl_{p}$, and α=1.11 and ν=0.56 are fitting (interpolation) parameters [21]. The threshold force reduces to the classical Euler form for an athermal elastic rod with $l_{p}\to \infty$.

So far we only discussed the physics of semiflexible filaments with a fixed contour length *L*, which was the basis of the WLC statistical model. However, especially at large tension forces, the real filament may be elastically stretched by extending the bonds holding its monomers in a chain. In actin filaments, these bonds are the physical interactions (hydrophobic and hydrogen bonds) holding the G-actin units together, with a characteristic energy of ∼25–30 $k_{B}T$; a similar bonding energy is found to hold the *β*-sheet peptides in an amyloid filament [23,24]. A stretched DNA double helix would elongate by modifying hydrogen-bond interactions between base pairs. In all of these cases, a finite stretching modulus was indeed detected by experiment.

When a semiflexible filament is highly stretched, its end-to-end factor will be close to unity, and an approximation can be implemented in Equation (8): $1-x^{2}≃2\left(1-x\right)$, making the force-extension relation at $x>x_{0}$:

(14) $$\begin{array}{ccc} f(x) & \approx & -\frac{\pi^{2}k_{B}Tl_{p}}{L^{2}}+\frac{k_{B}T}{\pi l_{p}{\left(1-x\right)}^{2}}\equiv -f_{c}+\frac{k_{B}T}{\pi l_{p}{\left(1-x\right)}^{2}}. \end{array}$$

An effective spring constant in charge of the elasticity of a filament can be defined by $K_{e}=df/Ldx$, producing

(15) $$\begin{array}{c} K_{e}=\frac{2k_{B}T}{\pi Ll_{p}{\left(1-x\right)}^{3}}\equiv k_{e}\sqrt{{\left(1+f/f_{c}\right)}^{3}}, \end{array}$$

where the second version of the expression is obtained implementing the Equation (14), with the shothand notation for the constant $k_{e}=2\pi^{7/2}k_{B}Tl_{p}^{2}/L^{4}$, and the Euler critical force $f_{c}$. If in addition, the material making up the filament has its own intrinsic Young’s modulus *Y*, an effective mechanical spring constant has to be added: $k_{m}∼Ya^{2}/L$, with *a* as radius of the filament. For actin filament of length 10μm, this spring constant is about 5 pN/nm, while a microtubule of the same length has a spring constant around 15 pN/nm. Considering the possibility of filament extension, the full length now becomes $L_{\mathrm{eff}}=L+f/k_{m}$ under a large tension *f*, and force-extension relation (with the approximation that *x* is close to 1) can be expressed as

(16) $$\begin{array}{ccc} x & = & \left(1+\frac{f}{k_{m}L}\right)\left[1-\sqrt{\frac{k_{B}T}{\pi l_{p}\left(f+f_{c}\right)}}\right]. \end{array}$$

In other words, the mechanic response becomes softer than entropic one if the filament is experiencing a very large stretching force, while the entropic mode is softer if a filament is stretched by a smaller force. Similar expressions were obtained by Odijk [17], Lipowsky et al. [25], and Holzapfel and Ogden [26].

**Figure 4:**
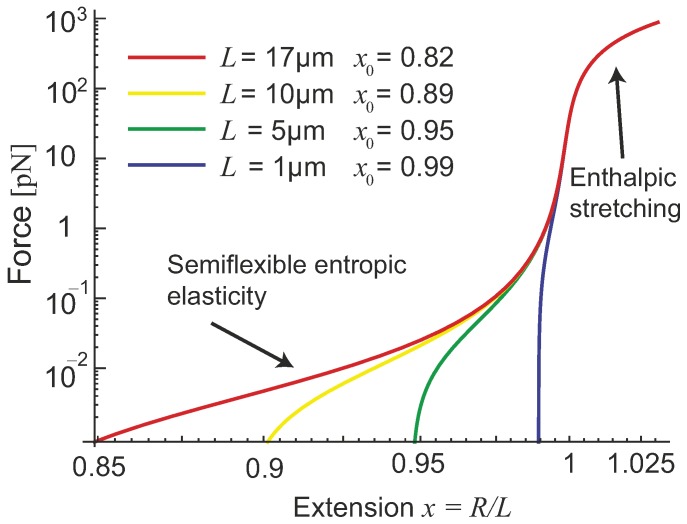
The comparison of the entropic (bending) and the tensile elasticity effects in the effective stretching modulus of a filament. Logarithmic force-extension curves show the response of extensible filaments using Equation (16). Parameters correspond to those of an actin filament of the persistence length $l_{p}=17\;\mu$m, Young modulus Y=2 GPa, filament diameter 7 nm, and several values of natural length *L* labelled in the plot. The crossover regime starts at $f_{c}$, when the ’linear-entropic’ response becomes ’nonlinear-entropic’ (as in Figure 3). The width of this crossover regime is determined by the ratio of $k_{m}/k_{e}$ (see text), after which the ’linear-mechanical’ (enthalpic) regime dominates, with the elastic modulus $k_{m}$.

There is a crossover region bridging the entropic stretching regime with the mechanical stretching regime, see Figure 4. One can make an analogy of the extensible semiflexible filament to two elastic springs connected in series, giving the effective spring constant K(f) as:

(17) $$\begin{array}{c} \frac{1}{K(f)}=\frac{1}{k_{e}\sqrt{{\left(1+f/f_{c}\right)}^{3}}}+\frac{1}{k_{m}}. \end{array}$$

If the tensile stretching force is small, i.e., $f<f_{c}$, the spring constant of the filament is approximately a constant $K_{0}=1/\left(k_{e}^{-1}+k_{m}^{-1}\right)$, which defines linear entropic response regime. If the tensile force is very large, $f>f_{c}{\left(k_{m}/k_{e}\right)}^{2/3}$, the filament extension is dominated by the mechanical (enthalpic) stretching, while the entropic elasticity essentially diverges. If the width of ’nonlinear-entropic’ regime is sufficiently large, the entropic part in Equation (17) scales as $f^{3/2}$; however, if the filament material is more easily stretched, the crossover could be narrow and much less distinct.

## 3. Network of Crosslinked Filaments

**Figure 5:**
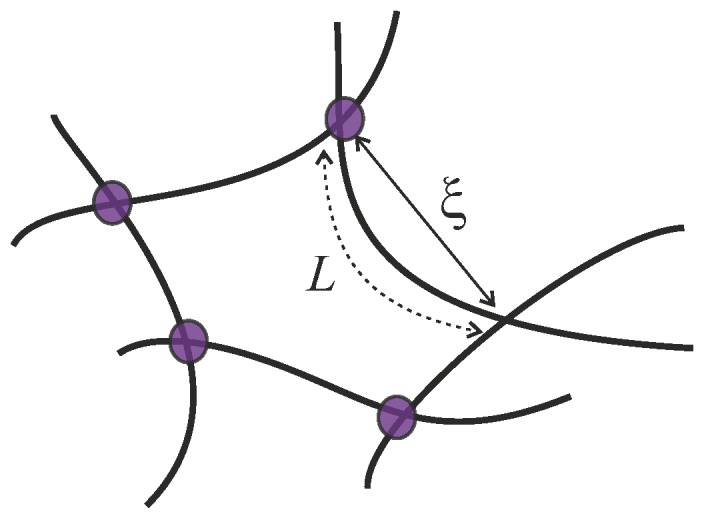
A sketch of a crosslinked filament network, showing the filament contour length *L* and the end-to-end distance *R*, which is equivalent to the mesh size *ξ* used later in the text.

Semiflexible filaments can be crosslinked together to form filament bundles and filament networks as illustrated in Figure 5. In biological context, the crosslinks are frequently various biological motors, for example, myosin and kinesin. Though physical properties of an individual semiflexible filament are relatively well studied, understanding how a semiflexible filament bundle or a network responds to an applied deformation is not a trivial question. For bundles, there is a wormlike bundle (WLB) model, proposed and developed by Heussinger et al. [27,28], where the main idea is that the free energy of a semiflexible bundle not only includes bending and stretch energy of the filaments, but also shear energy due to sliding of neighboring filaments with respect to each other. This model works well with the filament bundles which possess a hierarchical structure, such as microtubules. Filament networks, with a more complex structure than that of an individual chain or a hierarchical bundle, show various emergent phenomena, such as negative normal stress of a sheared network or marginal stability of a filament network with reduced number of crosslinks. Compared with the widely used WLB model for semiflexible bundles, there are different approaches for a filament network, which are constructed in various ways. In this section we will mainly focus on reviewing models on filament networks.

### 3.1. Models of the Filament Network Elasticity

*Storm et al. model* [29]. Storm et al. proposed a theoretical model of equilibrium semiflexible network, where all the filaments are assumed to be deformed affinely. Suppose that E is the deformation tensor, and $\rho ∼1/\xi^{2}$ is the filament length per unit volume (which is a measure of network density). After deformation, the length density of the filaments per unit volume crossing a plane perpendicular to *j* axis changes to $\rho E_{jk}n_{k}/\det E$, where n is the orientation of a filament end-to-end vector in an initially undeformed network, and detE measures the relative volume change of the deformed network. The tension acting on the filament bridging two neighboring crosslinks can be denoted by f(|E·n|−1), where |E·n|−1 represents for the axial strain of the filament, and the specific form of the tension force can be obtained from the preferred force-extension relationship discussed in previous section. The *i*th component of the tension can be calculated as $f\left(|E\cdot n|-1\right)E_{il}n_{l}/\left|E\cdot n\right|$, and the *ij* component of the stress tensor can be obtained by following the Doi-Edwards construction of adding all contributions of the filaments weighed by the filament length crossing the *j* plane [1]:

(18) $$\begin{array}{ccc} \sigma_{ij} & = & \frac{\rho }{\det E}\langle f\left(|E\cdot n|-1\right)\frac{E_{il}n_{l}E_{jk}n_{k}}{\left|E\cdot n\right|}\rangle . \end{array}$$

A simple application of the theory is for the simple shear, where the sample experiences a deformation:

(19) $$\begin{array}{c} E=\left(\begin{array}{ccc} 1 & \gamma & 0\\ 0 & 1 & 0\\ 0 & 0 & 1 \end{array}\right), \end{array}$$

where *γ* is shear strain. In the case of small shear strain (linear elastic response), the stress defined in Equation (18) will be simplified as [30]:

(20) $$\begin{array}{ccc} \sigma_{ij} & = & \rho \langle f\left(\gamma n_{x}n_{y}\right)n_{i}n_{j}\rangle +O\left(\gamma^{3}\right), \end{array}$$

where detE=1 for the simple shear. Mackintosh et al. [18] applied the linear regime of the force-extension relation provided in Equation (11), which makes the xy component of shear stress:

(21) $$\begin{array}{c} \sigma_{xy}=\rho k_{B}T\frac{90l_{p}^{2}}{L^{3}}\gamma \langle n_{x}n_{y}n_{x}n_{y}\rangle . \end{array}$$

Both here and in Equation (18) the averaging is over the statistical ensemble of filaments, having random orientations in the network. The shear modulus of a filament network consisting of isotropically distributed filaments can be obtained as $G_{0}=6\rho k_{B}Tl_{p}^{2}/L^{3}$, as a linear function of crosslink density. For a given concentration of monomer in the system $c\approx 1/a\xi^{2}$, the distance to find one chain to be intersected with another, or the distance between two neighboring entanglements, is calculated to be about $L∼l_{p}^{1/5}{\left(ac\right)}^{-2/5}$, which gives the scaling of the shear modulus $G_{0}∼k_{B}Tl_{p}^{7/5}{\left(ac\right)}^{11/5}$ [18]. There is usually a linear regime under small deformation and a nonlinear regime under large deformation in the elasticity of a filament network. By defining the crossover strain and stress from the linear regime to the nonlinear one as $\gamma_{t}=L/6l_{p}$ and $\sigma_{t}=\rho k_{B}Tl_{p}/L^{2}$, respectively, one can obtain the relationship between the linear shear modulus and the crossover stress as $c^{1/2}G_{0}∼\sigma_{t}^{3/2}$, recalling that ρ∼c [31].

**Figure 6:**
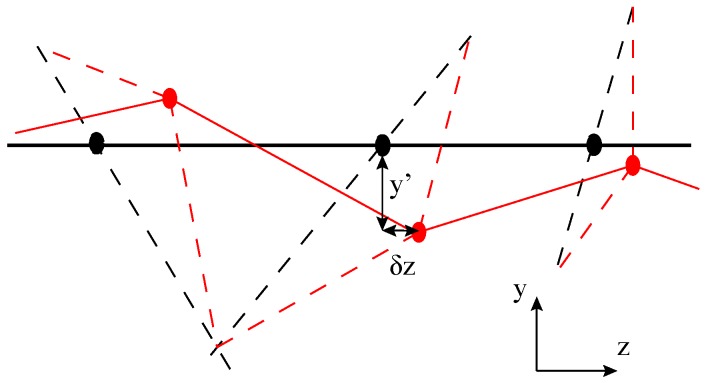
Nonaffine deformation by axial displacement δz of the primary filament (drawn as a solid line, horizontally) and subsequent transverse deflection $y^{\prime }$ of the crosslinks to restore the segment lengths of the other filaments (dashed lines), to first order in δz). Circles are crosslinks before and after deformation.

*Heussinger-Frey model* [32,33]. In contrast to perfect crystalline solids or the isotropic homogeneous rubbery networks, in filament networks the non-affine deformations cannot strictly be avoided. That is, network local structure would relax on the scale of a single mesh unit to lower the local energy and achieve global equilibrium. In 2006, Heussinger and Frey proposed a theoretical model for semiflexible filament network where the filaments experience non-affine deformations, which is also named as floppy mode model (sketched in Figure 6).

Assume a 2D filament network with area *A*, which consists of *N* filaments with uniformly distributed orientations and positions. The isotropic distribution of angles between two intersecting filaments gives P(θ)=sinθ/2, and the random distribution of the mesh size (taken as approximately equal to the contour length for a stiff filament connecting two neighboring crosslinks) can be written as P(ξ)=exp[−ξ/〈ξ〉]/ξ. Here the average mesh size can be related to the line density as $\langle \xi \rangle =\pi /2\rho =\pi A/2L_{f}N$, where $L_{f}$ is the full length of a filament, rather than the distance between two neighboring crosslinks. For subfilaments with radius *a*, which connect two neighboring crosslinks, they have a high stretching stiffness $k_{∥}=4\kappa /\xi a^{2}$, and a relatively softer bending stiffness $k_{⊥}=3\kappa /\xi^{3}$, using the measure of native filament bending modulus $\kappa =k_{B}Tl_{p}$. From numerical simulations by Wilhelm and Frey [34], it was found that the effective shear modulus of such a network has two distinct regimes, characterized by a length scale $l_{c}=L_{f}{\left(\delta \rho L_{f}\right)}^{-\nu }$ with ν≈2.84. For filaments with radius $a\gg l_{c}$, the network responds affinely and the elastic response is mainly arises from the stretching deformation, and the shear modulus $G_{aff}∼k_{∥}$ independently of the total filament length; for filaments with radius $a\ll l_{c}$, the non-affine bending deformations dominates in the mechanic response of the network, and the shear modulus depends on the total fiber length as $G_{na}∼k_{⊥}\left(\langle \xi \rangle \right){\left(L_{f}/\langle \xi \rangle \right)}^{\mu -3}$ with μ≈6.67.

In Heussinger-Frey model, the filament is relatively long compared with the mesh size, $L_{f}>\xi >a$, i.e., a filament can be shared in several meshes. A subfilament of length *ξ* connecting two crosslinks typically stores the energy $w_{b}\left(\xi \right)≃\kappa \delta_{na}^{2}/\xi^{3}≃\kappa L_{f}^{2}/\xi^{3}$. Then the soft bending response is incorporated, in charge of the local relaxation of one subfilament with respect to its neighbors, and the average bending energy stored in a whole filament consisting *n* subfilaments (line density defined as $\rho =n/L_{f}$) can be expressed as

(22) $$\begin{array}{c} \langle W_{b}\rangle ≃n\int_{l_{min}}^{\infty }d\xi P\left(\xi \right)\frac{\kappa \delta_{na}^{2}}{\xi^{3}}, \end{array}$$

where $\delta_{na}$ is the non-affine displacement which is assumed to scale as the total length of the filament (the affine displacement is on the length scale of the mesh size), and $l_{min}$ is introduced as the cutoff as the mesh with small size does not contribute to the bending energy obviously, due to their high bending stiffness $k_{⊥}∼\xi^{-3}$. In other words, the filaments with length smaller than $l_{min}$ will relax from their floppy mode deformation $\delta_{na}$, which reduces their bending energy from $w_{b}\left(l_{min}\right)$ to zero. However, this type of relaxation leads to the movement of the whole filament, which results in a floppy mode of the whole filament. By equalizing $w_{b}\left(l_{min}\right)$ to the average bending energy $\langle W_{b}\rangle$, $l_{min}≃1/\rho^{2}L_{f}$ and $\langle W_{b}\rangle ≃\kappa /L_{f}{\left(\rho L_{f}\right)}^{6}$ are obtained. Moreover, the shear modulus $G_{na}≃\rho /L_{f}\langle W_{b}\rangle ∼\rho^{7}$, which closely coincides with the simulation result as $\rho^{6.67}$. The crossover component ν=3 can be obtained by equalizing bending and stretching energy $\langle W_{b}\rangle =\langle W_{s}\rangle ≃\kappa L_{f}/r^{2}$.

### 3.2. Continuum Models of Semiflexible Filament Network

**Figure 7:**
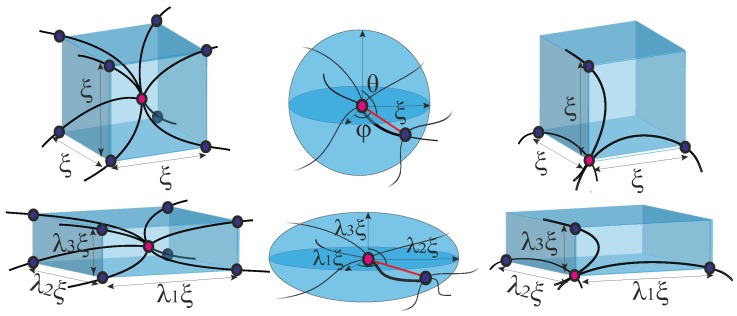
Cells in a semiflexible network before and after deformations for body-centred cubic lattice of 8-chain model, the homogeneous sphere in 1-chain model, and primitive cubic lattice of 3-chain model.

For rubber elasticity [35,36], there is a widely used class of theories formulated by constructing an effective continuum composed by periodically repeating blocks. In these models the free energy of the network can usually be explicitly expressed as a function of the invariants of the deformation tensor E: $I_{1}=\lambda_{1}^{2}+\lambda_{2}^{2}+\lambda_{3}^{2}$, $I_{2}=\lambda_{1}^{2}\lambda_{2}^{2}+\lambda_{2}^{2}\lambda_{3}^{2}+\lambda_{3}^{2}\lambda_{1}^{2}$ and $I_{3}=\lambda_{1}^{2}\lambda_{2}^{2}\lambda_{3}^{2}$, where $\lambda_{1,2,3}$ are the eigenvalues of E (see Figure 7 for illustration). Such continuum models are often named unit-cell models, where the cells, or the repeating blocks, are imaginary spheres for 1-chain model, cubes for 3- and 8-chain models, or tetrahedra for the 4-chain model. The macroscopic elastic free energy of the network is obtained by multiplying the free energy of an individual cell by the number of cells, whereas any non-affinity is assumed to be confined within a cell. Similarly, based on the energy of an individual filament as discussed above, several continuum models of semiflexible filament network are proposed.

*8-chain model.* In 2008, Palmer and Boyce [20] applied the 8-chain model for a filament network, where the body-centered cubic cell is constructed with eight filaments connected from the center to all eight lattice points in the corners. The lengths of the edges of the cell without any deformation are equal to *ξ*, as we define the mesh size, while the connecting chains are shorter by a factor $\sqrt{3}/2$. The important feature of the 8-chain model is that on deformation all eight chains in the cubic cell are stretched in exactly the same way, with the length changing from $\sqrt{3}\xi /2$ to simply $\sqrt{I_{1}}\xi /2$, since the cell is aligned along the principal axes of deformation tensor E by construction. Then the free energy density of the network is given directly by summing all equivalent single-filament expressions:

(23) $$\begin{array}{ccc} F_{8c}\left(I_{1}\right) & = & nF_{\mathrm{chain}}\left(\sqrt{I_{1}/3}\xi \right). \end{array}$$

where *n* is the number of crosslinked filament segments per unit volume, and the explicit energy form of an individual filament, $F_{\mathrm{chain}}$, can be chosen from the models introduced in the previous section. Though 8-chain model succeeds in fitting stress-strain relationship in sheared filament networks, as illustrated in next section, it fails to explain negative normal stress due to the intrinsic isotropy in the 8-chain model. The negative normal stress, as well as other important effects such as the marginal stability, are significant physical properties that need to be addressed.

*1-chain model.* In 2013, Unterberger et al. [37] applied the 1-chain model to describe the equilibrium elasticity of the filament network. In this model, one end of a filament is fixed at the center of an imaginary spherical cell, while the other end is on the sphere surface with an arbitrary, isotropic orientation (θ,φ). When deformed (with the deformation tensor E), the sphere will be deformed into an ellipsoid, where the lengths of the three semi-axes are $\xi \lambda_{1}$, $\xi \lambda_{2}$ and $\xi \lambda_{3}$, respectively. The elastic energy density of the 1-chain network model can be expressed as an orientational average of a deformed filament,

(24) $$\begin{array}{c} F_{1}c=n\int \frac{\sin\theta d\theta \;d\varphi }{4\pi }F_{\mathrm{chain}}\left[\tilde{\lambda }\left(\theta ,\varphi \right)\;\xi \right], \end{array}$$

where $\tilde{\lambda }\left(\theta ,\varphi \right)=\sqrt{\sin^{2}\theta \left(\cos^{2}\varphi \lambda_{1}^{2}+\sin^{2}\varphi \lambda_{2}^{2}\right)+\cos^{2}\theta \lambda_{3}^{2}}$ is the deformation ratio at orientation (θ,φ). If taking the ’entropic spring’ limit of the Gaussian chain, the average $F_{1}c$ reduces to the neo-Hookean model for rubber, expressed as $nk_{B}T\left(2\xi^{2}/3\pi l_{p}L\right)\left[\lambda_{1}^{2}+\lambda_{2}^{2}+\lambda_{3}^{2}\right]$, with the prefactor as the shear modulus, which is the basis of the classical rubber elasticity. However, with the more complicated filament deformation energy, the lack of an explicit form of $F_{1}c$ as a function of the strain invariants limits the applications of 1-chain model.

*3-chain model.* In 2016, Meng and Terentjev [38] applied the 3-chain model, where an imaginary cubic cell is constructed with the lattice points representing the crosslinking sites, and the edges aligned along the principle directions of the deformation tensor E. Three orthogonal chains at each lattice point in one cell are linked with their end-to-end vectors along the edges, and the equilibrium mesh size is *ξ*. On deformation, the lengths of three perpendicular edges at one lattice point become $\lambda_{1}\xi$, $\lambda_{2}\xi$ and $\lambda_{3}\xi$, respectively. Then the free energy density of such a network can be explicitly expressed as a function of the strain invariants by applying the free energy form of an individual filament defined in Equation (8):

(25) $$\begin{array}{c} F_{3c}\left(\left\{\lambda_{i=1,2,3}\right\}\right)=\frac{n}{3}\sum_{i=1,2,3}F_{\mathrm{chain}}\left(\lambda_{i}\xi \right)=\frac{nk_{B}T}{3}\left[\pi^{2}c\left(3-x^{2}I_{1}\right)+\frac{3-2I_{1}x^{2}+I_{2}x^{4}}{\pi c\left(1-I_{1}x^{2}+I_{2}x^{4}-I_{3}x^{6}\right)}\right], \end{array}$$

where $c=l_{p}/2L$ and x=ξ/L, as used in Equation (8). When the elastic energy is expressed as a function of strain invariants $I_{1,2,3}$, the stress tensor of an incompressible material (with $I_{3}=1$) can be obtained as [39,40]:

(26) $$\begin{array}{ccc} \sigma_{ij} & = & 2\left[\left(\frac{\partial F}{\partial I_{1}}+I_{1}\frac{\partial F}{\partial I_{2}}\right)C_{ij}-\left(I_{1}\frac{\partial F}{\partial I_{1}}+2I_{2}\frac{\partial F}{\partial I_{2}}\right)\frac{\delta_{ij}}{3}-\frac{\partial F}{\partial I_{2}}C_{ik}C_{kj}\right]-P\delta_{ij}, \end{array}$$

where $C=EE^{T}$ is the left Cauchy strain tensor, and *P* the Lagrangian multiplier for incompressibility, the value of which determined by the boundary conditions. This is also known as constitutive relationship, or stress-strain relationship. Given the compact analytical form of the elastic energy Equation (25), the equilibrium stress-strain relation can now be derived for any applied deformation.

### 3.3. Applications: Simple Shear, Network Stability and Negative Normal Stress

Consider the simple shear defined in Equation (19), i.e., a deformation when a point in Cartesian coordinates (x,y,z) in an original material will change to (x+γy,y,z) after being sheared with shear strain *γ*. The incompressibility is satisfied automatically in this deformation, with $I_{3}=1$ and the other two strain invariants $I_{1}=I_{2}=3+\gamma^{2}$. The shear stress obtained from the general constitutive relation (26) of the 3-chain and the 8-chain model are, respectively [38]:

(27) $$\begin{array}{ccc} {\left[\sigma_{xy}\right]}_{3c} & = & \frac{2}{3}nk_{B}T\gamma x^{2}\left[\frac{\left(1-x^{4}\right)}{c\pi {\left[1-\left(2+\gamma^{2}\right)x^{2}+x^{4}\right]}^{2}}-c\pi^{2}\right], \end{array}$$

(28) $$\begin{array}{ccc} {\left[\sigma_{xz}\right]}_{8c} & = & \frac{2}{3}nk_{B}T\gamma x^{2}\left[\frac{9}{c\pi {\left[3-\left(3+\gamma^{2}\right)x^{2}\right]}^{2}}-c\pi^{2}\right]. \end{array}$$

**Figure 8:**
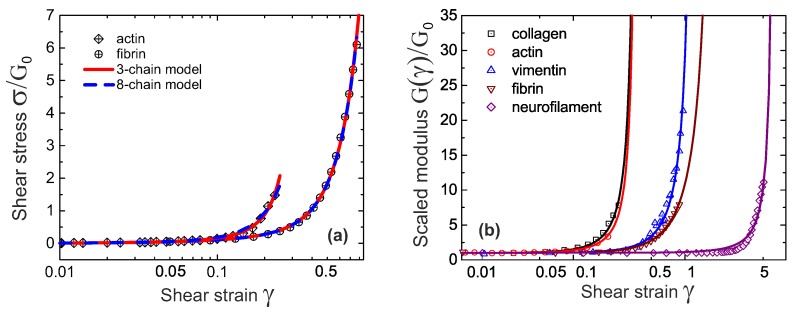
(**a**) Shear stress-strain relation fitted by 3-chain and 8-chain model (data from Ref. [29,41]); (**b**) Fitting modulus-strain data from Ref. [29] of sheared collagen, actin, vimentin, fibrin and neurofilament.

Both 3-chain and 8-chain models fit the shear-experiment data from Refs. [29,41] equally well, as shown in Figure 8a. We will need more stringent tests to distinguish which of these two models (both offering compact analytical expressions) is working better in filament networks. The stiffness, *c*, and the initial end-to-end factor, x=ξ/L, obtained by fitting with 3-chain model are a bit smaller than those in 8-chain model, but both in the semiflexible regime. Note that in rubber elasticity, there are two types of shear modulus, the nominal shear modulus $G\left(\gamma \right)=\sigma_{xy}\left(\gamma \right)/\gamma$, and the differential shear modulus $K\left(\gamma \right)=\partial \sigma_{xy}/\partial \gamma$ [19,29]. The linear shear modulus of the network, $G_{0}$, is equal to the differential modulus *K* for γ→0.

Intrinsic heterogeneity in the 3-chain model, reflected in the fact that the filament lying along the maximum principle stretch direction dominates the response of the whole cubic in semiflexible networks (due to the high nonlinearity of the filament tensile elasticity), is supposed to be closer to the realistic physics of filament network. In Figure 8b, experimental data for a wide variety of semiflexible filaments is fit by Equation (27). Fitted parameters (c,x) in Table 1, along with other parameters for different filament network coincides with those reported in experiments. An interesting point is that by calculating $x_{0}=\sqrt{1-c^{*}/c}$ for different filaments listed in Table 1, it is found that the fitted *x* is close to, but slightly smaller than $x_{0}$, indicating all of the filaments in Figure 8 are very slightly pre-compressed, rather than pre-stretched in the equilibrium state of the network.

As shown in Table 1, the ratio of fitted parameters $c/x=l_{p}/2\xi$ remains close to 1, for these semiflexible filaments with various effective stiffness. Moreover, it will become clear that all the networks we examined here lie very near the stability boundary, when discussing about the network stability (Table 1). It remains as a problem whether the fact that the mesh size is close to the filament persistence length is an unintended result of different crosslinking density in experiments [29], or is a relevant and universal biological feature.

**Figure 9:**
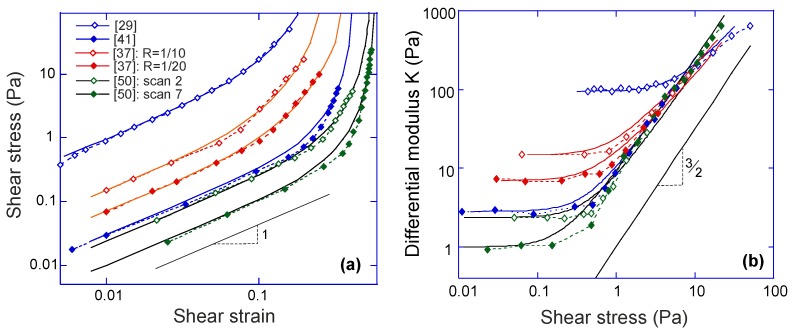
(**a**) Fitting stress-strain experimental data of different actin networks under simple shear (the source references labelled in the plot). The log-log plot nature highlights the linear-elasticity regime with the modulus $G_{0}$, before the stiffening sets in at higher shear; (**b**) The stress-stiffening of actin networks represented by the $K∼\sigma^{3/2}$ scaling relation. All data sets are the same as labelled in the plot (a), and solid lines are theoretical curves using the same fitted parameters.

An important scaling law in sheared filament network is about its differential modulus and the shear stress, given by $K∼\sigma^{3/2}$, which was found in a number of experiments and theories [31,41,46,47,48,49]. In 3-chain model, the differential shear modulus K(γ) can be expanded in powers of shear strain *γ*:

$$\begin{array}{c} K\left(\gamma \right)=\frac{\partial \sigma_{xy}}{\partial \gamma }\approx \frac{2}{3}nk_{B}Tx^{2}\left[\frac{1+x^{2}}{\pi c{\left(1-x^{2}\right)}^{3}}-\pi^{2}c\right]+4nk_{B}Tx^{4}\frac{1+x^{2}}{c\pi {\left(1-x^{2}\right)}^{5}}\;\gamma^{2}. \end{array}$$

where the first term represents the linear shear modulus $G_{0}$ in charge of the linear response, and the second term represents the first strain-dependent correction, or the shear-hardening part of the modulus. The crossover between the linear and nonlinear response is obtained when these two terms are comparable with each other. In the limit of a stiff network with x→1, the crossover, or transition shear strain can be approximated as $\gamma_{t}∼\left(1-x^{2}\right)/\sqrt{6}$, and the corresponding stress is $\sigma_{t}=G_{0}\gamma_{t}∼2\sqrt{2}nk_{B}T/\left[3\sqrt{3}c\pi {\left(1-x^{2}\right)}^{2}\right]$, see Figure 9a. This stress $\sigma_{t}$ increases with the temperature as ${\left(k_{B}T\right)}^{2}/\kappa$, which coincides with the observations in [49].

When the shear strain approaches the divergence point, i.e., γ→1/x−x in Equation (27), the stress and the differential modulus can be approximated as,

(29) $$\begin{array}{ccc} \sigma (\gamma ) & ≃ & nk_{B}T\frac{2\gamma x^{2}\left(1-x^{4}\right)}{3\pi c{\left[1-\left(2+\gamma^{2}\right)x^{2}+x^{4}\right]}^{2}}, \end{array}$$

(30) $$\begin{array}{ccc} K(\gamma ) & ≃ & nk_{B}T\frac{2\left(1-x^{4}\right)\left[x^{2}-\left(2-3\gamma^{2}\right)x^{4}+x^{6}\right]}{3\pi c{\left[1-\left(2+\gamma^{2}\right)x^{2}+x^{4}\right]}^{3}}. \end{array}$$

Both expression diverge due to the same vanishing denominator, while maintaining the obvious scaling relation $K∼\sigma^{3/2}$. We see this limit exposed clearly in Figure 9b for several very different actin networks. In this plot, the data points are the same as in Figure 9a, and the predicted curves of K(σ) is obtained by differentiating Equation (27), while using parameters $G_{0},c$ and *x* fitted in Figure 9a, for each dataset. Note that this 3/2 scaling at large deformation, acting as an intrinsic property of filament network, arises from mechanic properties of an individual filament, as

(31) $$\begin{array}{c} \frac{d\sigma }{d\gamma }∼\frac{df}{dx}∼\frac{1}{{\left(1-x^{2}\right)}^{3}}∼f^{3/2}\;\mathrm{when}\;x\to 1. \end{array}$$

One can easily check it with the force-extension relations given in the previous section. Note that in many cases, such as in Unterberger et al. [37], the filament network retains transient activity, which results in stress-softening and plasticity at higher stress due to the breakage of the crosslinks (this is also the case in experiments [50] before the actin filaments formed bundles, or with F-actin crosslinked by filamin). This type of filament network with transient crosslinks will be discussed in the next section. The actual data for the actin network of Storm et al. [29] also deviates from the 3/2 scaling. In fact, the authors of [29] develop their own theory invoking various additional factors (e.g., filament extensibility) to account for this data. However, the basic 3-chain model, assuming permanent crosslinks, bulk incompressibility and inextensible filaments, evidently fits both σ(γ) and K(σ) data very well. This fact might indicate that, for these values of *c* and *x*, the final crossover to this 3/2 characteristic scaling regime would occur at an even higher stress (at which point the network would probably not survive in practice).

*Network stability and non-affine effects.* Although the filament network can be pre-compressed, force-free or pre-stretched at its reference state, i.e., $x<,=,\mathrm{or}>x_{0}$, there is a condition for having a mechanically stable network, given by the linear shear modulus $G_{0}\geq 0$. In the 3-chain model, using the expression for $G_{0}$ in Equation (29), this condition translates into:

(32) $$\begin{array}{c} c\leq \frac{1}{\pi^{3/2}}\frac{\sqrt{1+x^{2}}}{{\left(1-x^{2}\right)}^{3/2}}. \end{array}$$

**Figure 10:**
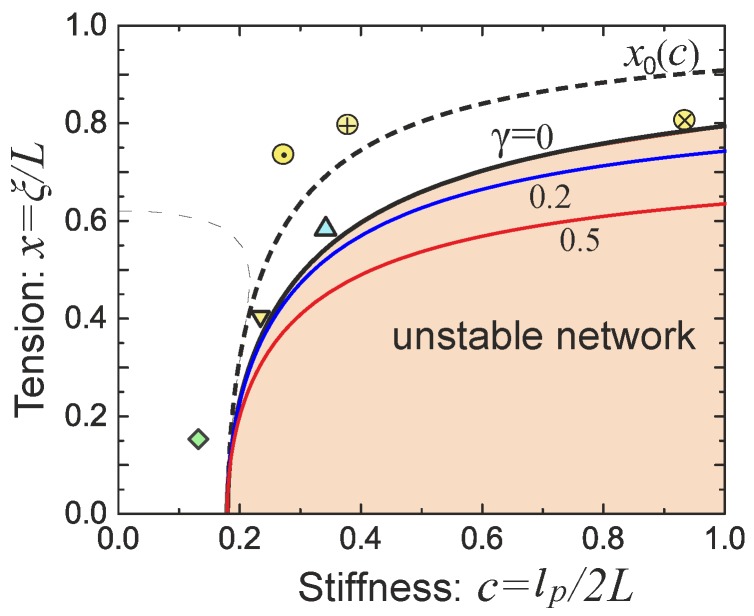
The map of network stability, in parameters of filament stiffness *c*, and and the filament pre-tension *x*, showing the boundary of network stability at several values of applied shear *γ*. The dashed line of the neutral (tension-free) filament $x_{0}\left(c\right)$ was defined in Section 2. Three ’softer’ filament networks from Table 1 are shown in this map: △ - vimentin, ▽ - fibrin, and ◇ - neurofilament networks. Three actin networks from Figure 9 also fit on this map: ⊕ - [41], ⊗ - [37], and ⊙ - [50].

Note that the right-hand side in Equation (32) is always greater than $c^{*}=1/\pi^{3/2}$, as defined in section 2 and illustrated in Figure 2b. Therefore, for sufficiently flexible chains with $c<c^{*}$, the network is always stable; indeed we always expect $G_{0}>0$ in the rubbery network. On the other hand, for filament networks composed of the more stiff filaments with $c\geq c^{*}$, the network strands have to be crosslinked with x=ξ/L exceeding the pre-tension threshold given by Equation (32), in order for the network to have a finite equilibrium shear modulus. In other words, there usually are forces acting on the crosslinked filaments in the network in order to be mechanically stable with a non-zero shear modulus. This notion is familiar from the “tensegrity” concept in biology and engineering [51]. For stiff athermal filament network, the window of pre-tension between the linear modulus $G_{0}=0$ at $x\approx 1-1/\pi {\left(2c\right)}^{2/3}$, and $G_{0}\to \infty$ at x→1 (assuming inextensible chains) is very narrow. The stability boundary of the network is plotted in Figure 10. The condition for $G_{0}=0$ gives the equilibrium case labelled as γ=0 in the phase diagram. The full analysis [38] shows that the magnitude of the shear strain modifies the stability condition, as represented by the additional marginal stability curves for γ=0.2 and 0.5. This shift means that the region of mechanical stability of filament network expands on increasing deformation, matching what the recent experimental data of Sharma et al. states [52].

As mentioned above, non-affine deformation in the local context of individual filament junctions and mesh cells, plays an important role when the mesh size is very large compared with the radius of the filament, or when the crosslink density is low. Note that in continuum models, such as 1-, 3-, or 8-chain model discussed here, the “mesh cells” are all deformed in the same way, but differently from the material, as these cells are constructed in orientation aligned with the principal stretching direction of the material, which is sometimes also referred to as “non-affinity”. But here we refer to the concepts of microscopic “affinity” or “non-affinity” for local deformation of individual filaments or mesh cells, rather than the global one between the orientation of a mesh cell and the macroscopic deformation of the material.

When the filament is compressed or stretched with a small deformation strain, the elasticity of the filament can be regarded as approximately linear. In other words, the filament in this case can be approximately as a linear spring with optimal end-to-end length as $\xi_{0}=L\sqrt{1-2L/\pi^{3/2}l_{p}}$ and spring constant $\alpha =2\pi^{7/2}k_{B}Tc^{2}$ from Equation (12), i.e., the free energy of the filament becomes approximately $\frac{1}{2}\alpha {\left(\xi -\xi_{0}\right)}^{2}$, where *ξ* is the end-to-end length of the filament. Note that in a crosslinked network, the length of the filament strand is not necessarily equal to the zero-force (optimal) one, which means the filament can be either pre-stretched, pre-compressed or remain force-free. In an isotropic material experiencing a small shear deformation, the affine part of the linear shear modulus can be calculated by Born-Huang theory as [53,54]:

(33) $$G_{0}=\frac{1}{V}\sum_{ij}\left(\alpha \xi_{e,\;ij}-f_{ij}\right)\xi_{e,\;ij}n_{x,\;ij}n_{x,\;ij}n_{y,\;ij}n_{y,\;ij},$$

where (ij) denotes for a bonded pair, $\xi_{e,\;ij}$ is the equilibrium end-to-end length of the filament bridging points (ij) in a network, $f_{ij}$ is the pre-tension force on the filament bridging the neighboring points, and $n_{ij}$ are unit vectors along the (ij) line. For such a filament, the force-extension relation gives $f_{ij}=\alpha \left(\xi_{e,\;ij}-\xi_{0,\;ij}\right)$. By substituting this linear force-extension relation back into Equation (33), the shear modulus takes the form $G_{0}=\left(zN_{c}/2V\right)\alpha \xi_{e}\xi_{0}\cdot \langle n_{x}n_{x}n_{y}n_{y}\rangle$, where $N_{c}$ is the number of crosslinks, and *z* is the number of nearest crosslinks connected with a chosen crosslink, or the average network connectivity. The averaging is over the statistical ensemble of orientations of bond vectors $n_{ij}$.

The non-affine part of the linear shear modulus reduces the modulus by the local force relaxation. Under the same assumptions of harmonic bonds, the non-balanced local relaxation force acting on a crosslink (i) can be expressed as [54] $\Xi_{i}=-\sum_{j}\left(\alpha \xi_{e,\;ij}-f_{ij}\right)n_{ij}n_{x,\;ij}n_{y,\;ij}$. In this case, the cancellation in the bracket leaves no pre-tension contribution and the local force only depends on the force-free distance $\xi_{0}$: $\Xi_{i}=-\sum_{j}\alpha \xi_{0,\;ij}n_{ij}n_{x,\;ij}n_{y,\;ij}$. The detailed derivation [55,56] gives the resulting non-affine part of the modulus: $G_{\mathrm{na}}=\left(3N_{c}/V\right)\alpha \xi_{0}^{2}\cdot \langle n_{x}n_{x}n_{y}n_{y}\rangle$. Combining these two parts, we obtain the estimate of effective linear shear modulus:

(34) $$G=G_{0}-G_{\mathrm{na}}=\frac{N_{c}}{V}\alpha \left(\frac{z}{2}\xi_{e}\xi_{0}-3\xi_{0}^{2}\right)\cdot \langle n_{x}n_{x}n_{y}n_{y}\rangle .$$

If chains are distributed isotropically in the network, then $\langle n_{x}n_{x}n_{y}n_{y}\rangle =1/15$ [55,56]. The important conclusion of this analysis is that the shear modulus is proportional to $G_{0}\left(1-6\xi_{0}/z\xi_{e}\right)$. That is, a connectivity of z≥6 is required in the network crosslinked with force-free filaments to be mechanically stable (a classical Maxwell criterion for the assumer central forces). Yet when the filaments are linked with pre-tension (i.e., $\xi_{e}>\xi_{0}$), the point of marginal stability becomes increasingly lower: $z\geq 6\xi_{0}/\xi_{e}$ (again, the “tensegrity” effect at play).

Note that in this schematic analysis of the role of non-affinity in filament network we used a random orientation of filaments as a basic assumption: strictly, $\langle n_{x}n_{x}n_{y}n_{y}\rangle =0$ for a 3-chain cubic cell, indicating that the 3-chain model cannot be used with nonaffine-effects in a simple way.

**Figure 11:**
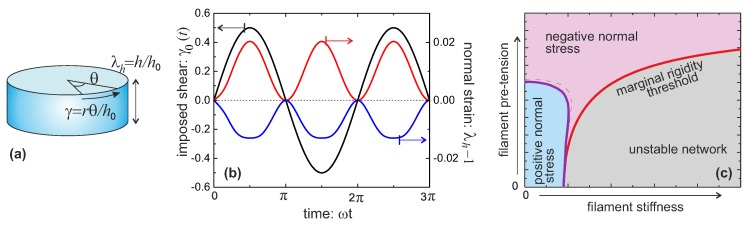
(**a**) Cylindric material under oscillating shear deformation; (**b**) The relation between oscillating shear strain *γ* at the outer surface of the cylinder (black curve) and the normal strain $\lambda_{h}-1$ for a flexible network (red curve) with c=0.1,x=0.1, and a stiff network (blue curve) with c=0.3,x=0.6; (**c**) Phase diagram of the network of different stiffness and filament pre-tension, showing the regions of positive/negative Poynting effect (normal stress) and also the boundary of network stability discussed earlier.

*Negative normal stress.* When being sheared, filament networks are usually found to show negative normal stress [57,58,59]. This is highly unusual and characteristic of the so-called auxetic materials, while the ordinary polymer networks (rubbers and gels) all show the positive normal stress, often visualized as the so-called Weissenberg effect. Most of the experiments until now reporting normal stress measurements in filament networks were conducted in the rotating cylindrical geometry of a standard rheometer, as shown in Figure 11a. Suppose the height and the radius of the undeformed cylinder are $h_{0}$ and $R_{0}$, respectively. When being deformed, they change to $h=\lambda_{h}h_{0}$ and $R=\lambda_{R}R_{0}$, with $\lambda_{h,R}$ as the stretch ratio along the longitudinal and the radial directions, respectively, with assumed incompressibility $\lambda_{h}\lambda_{R}^{2}=1$. In the cylindrical coordinate system, the point at (r,θ,z) has its position change to $\left(r/\sqrt{\lambda_{h}},\theta +\Theta z/h_{0},\lambda_{h}z\right)$, after rotating the top plate with angle Θ (pure shear, rather than simple shear, is used for discussions about the normal stress, because of the uncertain boundary conditions in simple shear case [60]), and the deformation tensor is written as,

(35) $$\begin{array}{c} E=\left(\begin{array}{ccc} 1/\sqrt{\lambda_{h}} & 0 & 0\\ 0 & 1/\sqrt{\lambda_{h}} & \gamma \left(r\right)/\sqrt{\lambda_{h}}\\ 0 & 0 & \lambda_{h} \end{array}\right), \end{array}$$

where $\gamma \left(r\right)=r\Theta /h_{0}$ is the shear strain, depending on the radial position in this parallel-plate geometry. In this case, the first two strain invariants are, $I_{1}=\lambda_{h}^{2}+\left[2+\gamma^{2}\right]/\lambda_{h}$ and $I_{2}=2\lambda_{h}+\left[1+\gamma^{2}\right]/\lambda_{h}^{2}$. Given the shear strain at the outermost surface, $\gamma_{0}=\gamma \left(R_{0}\right)$, the total free energy can be expressed as a function of the stretching ratio $\lambda_{h}$ along the *z* axis, after integration over radius,

(36) $$\begin{array}{c} F\left(\lambda_{h};\gamma_{0}\right)=\int_{0}^{R_{0}}drF_{3c}\left(\lambda_{h},r;\gamma_{0}\right). \end{array}$$

Then upon minimization of the free energy, the equilibrium $\lambda_{h}$ can be obtained [38]. When the cylinder radius becomes smaller (or larger) upon shear, with its height becoming correspondingly larger (or smaller) – this phenomenon is called the positive (or negative) Poynting effect [61,62]; this geometric effect with stress-free top plate corresponds to the positive (negative) normal stress required to maintain the fixed plate separation in a more common rheometry experiment [57,58].

Figure 11b shows the results for two model materials with different filament stiffness and pre-tension [38]. A flexible network (c=0.1,x=0.1) has a positive Poynting effect, or positive normal stress is required to counter the expansion along the height (the Weissenberg effect), where the mesh size is much smaller than the contour length of the subfilaments connecting the neighboring crosslinks ($x_{0}\ll 1$) thus the entropic energy is important. With the chains being stretched along the principal extension direction in the shear geometry of Figure 11a, the chains in other two principle directions are being compressed, leading to the material contraction along the radial and circumferential directions. Conversely, if the mesh size is comparable to the contour length of the subfilaments, $x_{0}\leq 1$, the material can behave in a negative Poynting effect manner, with contracted height and expanded radius, or exerting the negative normal stress is required to maintain the fixed height. This is because the force acting on the chains directed along the principal extension in Figure 11a is close to a divergence if $x_{0}\to 1$, and it causes less energy when the material is compressed in the longitudinal direction, rather than stretched. Similar reason works also for a network of more stiff filaments (see red curve for $c=0.3>c^{*},x=0.6$). The magnitude of normal stress in a fixed height geometry can be estimated from the linear relationship $\sigma_{h}∼3G_{0}\left(\lambda_{h}-1\right)$, which works for the case where the normal strain is small and the Young modulus is $3G_{0}$. Taking the fibrin in Table 1 as an example, the normal stress is about 11.5 Pa under a shear strain of $\gamma_{0}=0.5$. These are very close to the observations in experiments [57,58]. The magnitude of $\sigma_{h}≃20$ Pa for the same shear also matches the actin results obtained in [37].

Figure 11c gives the full ’phase diagram’ of the stiffness-pretension parameter space (c,x), with phase boundaries separating stable/unstable regions and positive/negative normal stress regions. There is a not shown weak dependence of this boundary on the magnitude of the applied shear strain, due to the inherent non-linearity of stress-strain response. Figure 11c shows that a sheared flexible network with $c<c^{*}$ usually shows a positive normal stress, while a semiflexible filament network has a negative negative normal stress, especially when the filaments are crosslinked with increasing pre-tension.

The above model explains the negative normal stress in equilibrium. de Cagny et al. [63] recently observed that the normal stress could be positive at short times after being sheared, and then becomes negative after a sufficient long relaxation time, which is dependent on the mesh size of the network. Clearly this question is yet far from being fully resolved.

## 4. Transient Filament Network with Breakable Crosslinks

**Figure 12:**
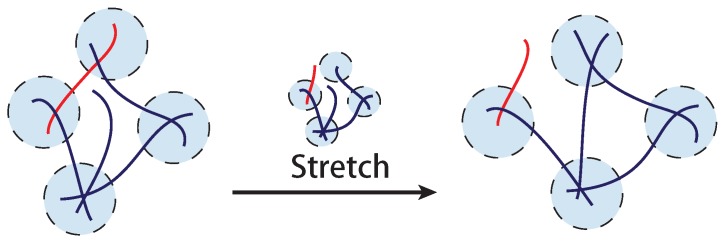
A sketch of a transient network where the filaments can be broken from, and re-crosslinked back to the network.

In contrast to a permanently crosslinked network such as classical rubber, biological crosslinks in a filament network can be dynamic. The classical collagen network is held together by hydrogen-bonded triple-helical segments that are known to dynamically respond to an applied tension. The F-actin network is often held together by molecular motors (such as myosins) or physically bonded proteins (such as filamins). Self-assembled biological filaments themselves can be broken from or re-crosslinked to the network. In synthetic networks, one frequently finds dynamic crosslinks formed by physical bonds (such as hydrophobic interactions or hydrogen bonds, Figure 12). Actually, the concept of a transient crosslink here is very similar to the “trap” defined in soft glassy systems [64,65], where semiflexible chains are assumed to be trapped in a glassy environment, and also to the dynamic “entanglement” in concentrated polymer solutions [1].

Due to the transient feature of such crosslinks, the resulting network shows characteristic rheological properties, such as a high stress relaxation at given constant strain, a yield stress upon ramp deformation, or the elastic-plastic transition. As transient filament network is a better analogy to the filament network in many biological systems, there are several relevant theories developed in the recent years from different viewpoints.

**Figure 13:**
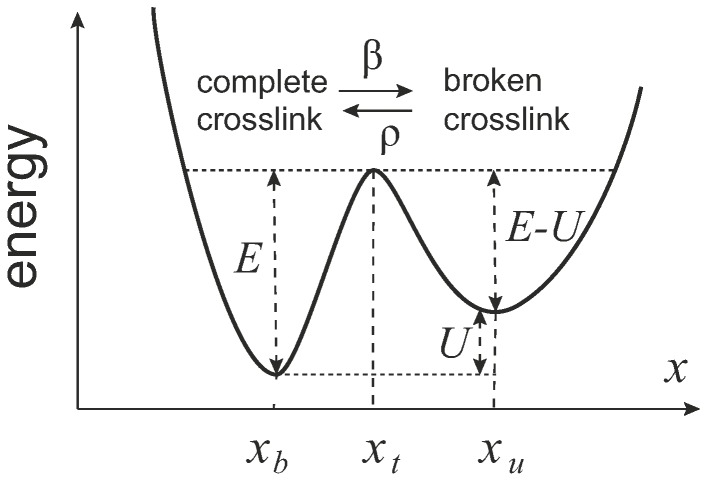
A sketch of effective interaction potential for complete and broken crosslinks in “glassy” filament network model.

In 2007, Kroy et al. proposed the glassy wormlike chain model [66,67], and then used it to study the viscoelastic properties of “sticky filament network” [68]. One assumes there is a certain crosslink breaking rate *β*, and also a rate of crosslink re-connection, *ρ*. In adiabatic approximation, the rates can be expressed by the activation laws (Figure 13):

(37) $$\begin{array}{c} \beta \tau_{0}=e^{\left[-E+\left(x_{t}-x_{b}\right)f\right]/k_{B}T},\;\;\;\rho \tau_{0}=e^{\left[-E+U-\left(x_{u}-x_{t}\right)f\right]/k_{B}T}, \end{array}$$

where $\tau_{0}$ is an intrinsic time scale of attempts due to the thermal fluctuations, *E* is the energy barrier for breaking the crosslink, *U* is the energy penalty for the broken crosslink, *f* is the pulling force, $x_{b}$ is the coordinate of crosslinked state, $x_{t}$ is the coordinate of the barrier, and $x_{u}$ is the coordinate of the uncrosslinked state (crosslink broken). The fraction of the complete crosslinks is interpreted via the effective change in filament length: $n\equiv L_{0}/L\left(t\right)$ (the fraction of broken crosslinks is 1−n), where $L_{0}$ is the minimum contour length of the filament between two crosslinks, and L(t) is the contour length of the filament between crosslinks at time *t*, which can change with the breakage and the formation of the crosslinks. Then the evolution equation of the fraction of the complete crosslinks is

(38) $$\begin{array}{c} \dot{n}\tau_{0}e^{E/k_{B}T}=-\left(e^{\left[U-\left(x_{u}-x_{t}\right)f\right]/k_{B}T}+e^{\left(x_{t}-x_{b}\right)f/k_{B}T}\right)n+e^{\left[U-\left(x_{u}-x_{t}\right)f\right]/k_{B}T}. \end{array}$$

Assuming that the linear shear modulus is proportional to the density of active crosslinks, the main difference in the complex modulus between the ordinary filament and the glassy semiflexible one in transient network is in the relaxation time $\tau_{n}$, which is a constant $\tau_{n0}$ in the first case, while for glassy semiflexible filament it depends on time via the L(t) relaxation:

(39) $$\begin{array}{ccc} \tau_{n} & = & \left\{\begin{array}{c} \tau_{n0},\;\;\;\;\;\;\lambda_{n}<L\left(t\right)\\ \tau_{n0}e^{E\left(\lambda_{n}/L\left(t\right)-1\right)/k_{B}T},\;\;\;\;\;\;\lambda_{n}\geq L\left(t\right) \end{array}\right., \end{array}$$

with $\lambda_{n}$ as wavelength of the *n*th mode. Microscopic complex modulus for transverse point excitations of a generic filament element was defined in this model as

(40) $$\begin{array}{c} g\left(\omega \right)=g^{\prime }\left(\omega \right)+ig^{\prime \prime }\left(\omega \right)=1/\left[\frac{L^{3}}{l_{p}\pi^{4}}\sum_{n=1}^{\infty }\frac{1}{\left(n^{4}+n^{2}f/f_{c}\right)\left(1+i\omega \tau_{n}\right)}\right], \end{array}$$

where *f* is filament tension, and $f_{c}$ the Euler buckling force for rigid rod as defined previously.

In 2008, Lieleg et al. [69,70] performed a rheological experiment on actin network crosslinked by rigor heavy meromyosins (HMM), which is yet another kind of transient crosslinks. They reported local stress relaxation and energy dissipation in an intermediate elasticity-dominated frequency regime. In order to explain this, they developed a semi-phenomenological model by introducing a breakage rate of the crosslinks, $\beta_{0}\approx 0.09\;s^{-1}$, which is a simpler version of the glassy filament network model above. By assuming a breakage rate of crosslinks, the number of the crosslinks decays exponentially with time as $N\left(t\right)∼N\left(0\right)\exp\left(-\beta_{0}t\right)$, which can be translated into the frequency space by Fourier transformation, $\hat{N}\left(\omega \right)$, where *ω* is the oscillation frequency in the experiment. With the rubber-elasticity relation G∝N, the storage and the loss modulus are written as functions of the oscillation frequency:

(41) $$\begin{array}{c} G^{\prime }\left(\omega \right)=G_{0}-a\frac{N\beta_{0}}{\beta_{0}^{2}/4\pi^{2}+\omega^{2}}+b{\left(\frac{\omega }{\omega_{0}}\right)}^{3/4}\;\;\;\mathrm{and}\;\;\;G^{\prime \prime }\left(\omega \right)=c\frac{N\omega }{\beta_{0}^{2}/4\pi^{2}+\omega^{2}}+d{\left(\frac{\omega }{\omega_{0}}\right)}^{3/4}, \end{array}$$

where the terms with fitting parameters *a* and *c* arise from the breakage of the crosslinks, via $\hat{N}\left(\omega \right)$, the $\omega^{3/4}$ terms with parameters *b* and *d* arise from the fluctuation of single filaments [30,71], and the time scale of the relaxation mode is set by $\omega_{0}$ which is dependent on the solvent viscosity. There is a characteristic frequency in experiments, $\omega_{\min}$, at which the dissipation has a minimum. When the frequency *ω* is larger than $\omega_{\min}$, the storage modulus is almost a constant, while decreasing at lower frequencies due to the breakage of crosslinks at longer time scales. This semi-phenomenological model provides a basic understanding in the viscoelastic response of a transient network at different time scales.

**Figure 14:**
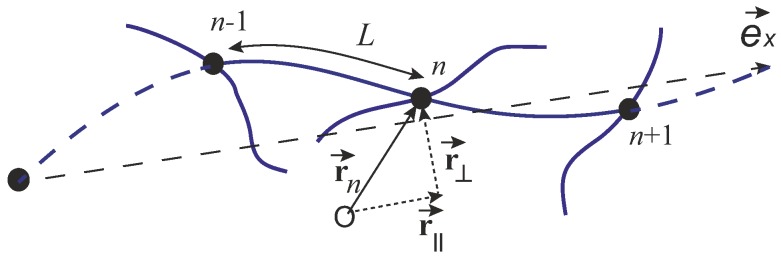
Sketch of an individual filament in a network of Broedersz et al. model [72].

In 2010, Broedersz et al. [72] proposed a model of transient filament networks based on crosslink-governed dynamics, where Monte Carlo simulations and an analytic approach are combined for explaining the rheological properties of the network. There are three time scales in the model: the thermal relaxation time for chains after breaking off a crosslink, $\tau_{\mathrm{eq}}$, the re-crosslink time, $\tau_{r}$, and the crosslink breakage time, $\tau_{b}$. In this model they are assumed to obey a relation: $\tau_{\mathrm{eq}}\ll \tau_{r}\ll \tau_{b}$. The elastic energy of the network can be expressed as a sum of the bending energy and the stretching energy of all the subfilaments connecting the pairs of crosslinks:

(42) $$\begin{array}{c} F=\frac{1}{L}\sum_{n}\left[\frac{\kappa }{2}{\left|\Delta t_{n}\right|}^{2}+\frac{\mu_{\mathrm{th}}}{2}{\left(|\Delta r_{n}|-L\right)}^{2}\right], \end{array}$$

where *L* is the length of a strand between the two crosslinks, which is assumed to be the same in the whole network, the sum is taken for all the crosslink positions $r_{n}$ (with the “mesh size” $\Delta r_{n}=r_{n+1}-r_{n}$), and $t_{n}$ is unit tangent vector of n*th* crosslink (sketched in Figure 14. In this case, the n*th* segment curvature is $|\Delta t_{n}|/L$, defining the first term (the bending energy). The second term in Equation (42) denotes for the elastic energy of the spring, where the stretching modulus $\mu_{\mathrm{th}}$ is directly related to the discussion below Equation (11) in Section 2. In order to calculate the polymer displacement due to the unbinding and rebinding, they separate the local equilibration step into a move to the mechanical equilibrium position, with an added stochastic contribution around this equilibrium. Mechanical relaxation step from the initial position $r_{n}^{i}$ to the local equilibrium position $r_{n}^{eq}$ is determined by

(43) $$\begin{array}{c} {\left.\frac{\partial F}{\partial r_{n}}\right|}_{r_{n}=r_{n}^{eq}}=0. \end{array}$$

Actually, this numerical method can describe non-affine deformation in filament networks. By solving Equation (43) discretely and taking the continuum long-wavelength limit, the leading order evolution equations with thermal fluctuations are

(44) $$\begin{array}{c} \tau_{b}\frac{\partial r_{∥}}{\partial t}=\frac{l_{c}^{2}}{2}\frac{\partial^{2}r_{∥}}{\partial x^{2}}+e_{x}\cdot \xi_{⊥},\;\tau_{b}\frac{\partial r_{⊥}}{\partial t}=\frac{l_{c}^{2}}{2}\frac{\partial^{2}r_{⊥}}{\partial x^{2}}+\xi_{⊥}. \end{array}$$

where *x* denotes for the position of the cross-linker along the average direction $e_{x}$ of the relaxed filament, and $r_{∥}$ and $r_{⊥}$ are the longitudinal and transverse deflections of the filament with respect to its average direction. The noise term $\xi_{⊥}$ possesses both thermal effects and also the local buckling contributions from thermal compression. Note that the noise actually depends on the local state of the polymer and couples the longitudinal and the transverse motion of a filament as described Equation (44). In the limit of small stretch modulus, $\mu \ll \mu_{\mathrm{th}}$, Equation (44) decouple and the resulting transverse contribution to the power spectrum ($C\left(\omega \right)=\langle |\delta l\left(\omega \right){|}^{2}\rangle$ with δl as extension of length) approaches $C_{⊥}∼\omega^{-7/4}$, in agreement with simulation. In this limit, the transverse bending dynamics are effectively those of a stiff filament fluctuating in a viscous solvent, which gives ${\langle |\delta l\left(t\right)|}^{2}\rangle ∼t^{3/4}$ [30,71]. Recently, this Langevin-type model was applied to active network systems [73].

**Figure 15:**
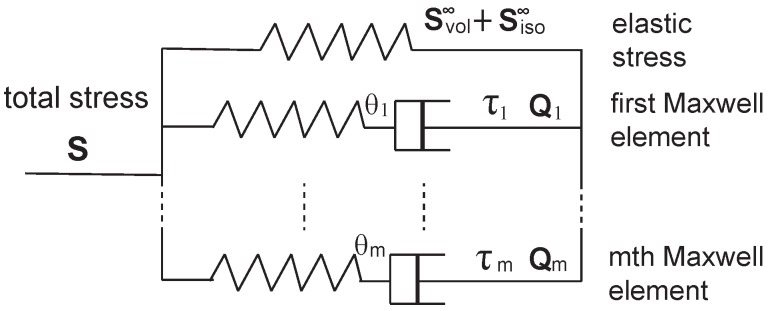
Schematic model of a viscoelastic material. The total stress S decomposes according to Equation (45). The spring in the first row represents the elastic stress, while the Maxwell elements contribute the non-equilibrium stresses $Q_{n}$,n=1,...,m. The mechanical properties of the springs and dashpots of Maxwell elements are defined by the free-energy parameters *θ* and the relaxation times *τ*, respectively.

In 2013, Unterberger et al. [74] applied a generalized Maxwell model to the transient filament network for describing its viscoelastic properties, as shown in Figure 15. In addition to the pure elastic part of the material, the model adds another *m* parallel Maxwell elements, each of which consists of an elastic spring and a viscous dashpots. In studies of viscoelasticity and relaxation, this is a common method of introducing the spectrum of relaxation times that is often necessary to describe complex relaxation processes.

Rather than using Cauchy stress σ, as defined in Equation (26), they applied the second Piola-Kirchhoff stress, S, which is related with Cauchy stress as: $S=\sqrt{\det C}\cdot E^{-1}\cdot \sigma \cdot E^{-T}$. There are three distinct contributions to the total stress of the material, which is expressed as

(45) $$\begin{array}{c} S=S_{vol}^{\infty }+S_{iso}^{\infty }+\sum_{n=1}^{m}Q_{n}. \end{array}$$

where the first term denotes the equilibrium volumetric part, the second term denotes the equilibrium isochoric-elastic part, and the third term reflects the isochoric relaxation (non-equilibrium) part. The first two contributions are typical elastic ones. Here we will only focus on the third part, which is closely related to the “transient” characteristics of the filament network. The volume-preserving non-equilibrium stress tensors $Q_{n}$, are defined as a function of internal variables, $\Gamma^{n}$, through the dissipative potential, $\Upsilon_{iso}$,

(46) $$\begin{array}{ccc} Q_{n} & = & {\left(\det E\right)}^{-2/3}\left(1-B^{-1}⊗B\right):\frac{2\partial \Upsilon_{iso}^{n}\left(\bar{B},\bar{\Gamma }\right)}{\partial \bar{B}}, \end{array}$$

where B is the right Cauchy tensor $B=E^{T}E$, $\bar{B}=\det E^{-2/3}B$ is the isochoric part of the right Cauchy tensor, and $\bar{\Gamma }$ is the isochoric part of the **Γ**. A set of differential equations and initial conditions for the non-equilibrium part of the *n*th Maxwell element, $Q_{n}$ is introduced to describe the transient property of the material, as

(47) $$\begin{array}{c} {\dot{Q}}_{n}+\frac{Q_{n}}{\tau_{n}}=\theta_{n}{\dot{S}}_{iso}^{\infty }\;,\;\;\;\mathrm{with}\;\;\;{\dot{Q}}_{n}\left(t=0\right)={\left[{\left(\det E\right)}^{-2/3}\left(1-B^{-1}⊗B\right):\frac{2\partial \Upsilon_{iso}^{n}\left(\bar{B},\bar{\Gamma }\right)}{\partial \bar{B}}\right]}_{t=0}, \end{array}$$

where $\theta_{n}$ are non-dimensional free energy parameters, related to the relaxation times $\tau_{n}$. Convolution integrals provided a solution for Equation (47) of the *n*th Maxwell element as

(48) $$\begin{array}{c} Q_{n}={\left.Q_{n}\right|}_{t=0}e^{-t/\tau_{n}}+\int_{0}^{t}dt^{\prime }e^{-\left(t-t^{\prime }\right)/\tau_{n}}\theta_{n}{\dot{S}}_{iso}^{\infty }\left(t\right), \end{array}$$

which basically describes how the energy of the material dissipates with time due to the transient crosslinks, and the time scale for the *n*th Maxwell element is given by its the relaxation time, $\tau_{n}$.

One could instead apply a dynamic theory of transient rubbery networks [75,76], which was based on classical Gaussian (neo-Hookean) chain response, to the case of filament network. This strategy is similar to the theory of equilibrium filament network, Equations (25) and (27), where the specific form of a non-linear elastic response of the filament strand was implemented in a continuum model. By combining the free energy form of a permanent filament network with the transient characteristics of its crosslinks, we could work out the time-dependent response to various modes of dynamic deformation.

We shall use the crosslink dynamics very similar with those in glassy wormlike filament network model, Equation (37). The rate of a chain to break from a crosslink depends on the force *f* acting on the chain as: $\beta =\beta_{0}e^{fa/k_{B}T}$, where $\beta_{0}$ is the breakage rate for a force-free chain, and *a* is the monomer size and taken as a characteristic length of a bond. As in Equation (37), $\beta_{0}\tau_{0}=e^{-E/k_{B}T}$. The dangling chains are able to re-crosslinked into the network with a rate *ρ*, determined by the barrier E−U (cf. Figure 13), although here we will assume that the free filament would be able to relax and re-crosslink in the force-free state (which is the basis of the stress relaxation in the material).

Suppose the total number of filaments in the network is $N_{\mathrm{tot}}$, with $N_{c}\left(0\right)$ of them crosslinked at the time t=0; $N_{b}\left(0\right)=N_{tot}-N_{c}\left(0\right)$ chains are un-crosslinked. The breakage rate of the crosslinks is a function of the local force acting on the given chain, rather than as a constant,and thus can be only defined locally and dynamically: it depends on both the current state at time *t* and the crosslinking state at time $t^{\prime }$ that defines the reference state for the given chain. We denote the breakage rate of the crosslinks at time *t* formed at time $t^{\prime }$ as $\beta_{t;t^{\prime }}$. After an infinitesimal time interval, Δt, the number of the originally crosslinked chains, will decrease to $N_{c}\left(0\right)\left[1-\beta_{0;0}\Delta t\right]≃N_{c}\left(0\right)\exp\left[-\beta_{0;0}\Delta t\right]$, where $\beta_{0;0}$ denotes the breakage rate at time t=0. Meanwhile, there will be $N_{b}\left(0\right)\rho \Delta t$ new filaments re-crosslinked into the network in the same time. By combining these two parts, the total number of the crosslinked chains at time t=Δt then becomes: $N_{c}\left(0\right)\exp\left[-\beta_{0;0}\Delta t\right]+N_{b}\left(0\right)\rho \Delta t$. After another time interval, i.e., at t=2Δt, the number of the crosslinked chains surviving from the beginning decreases further to $N_{c}\left(0\right)\exp\left[-\beta_{0;0}\Delta t-\beta_{\Delta t;0}\Delta t\right]$; the number of chains which were crosslinked during the first time interval decreases to $N_{b}\left(0\right)\rho \Delta t\exp\left[-\beta_{\Delta t;\Delta t}\Delta t\right]$; finally, the number of newly crosslinked chain during the second time interval is $N_{b}\left(\Delta t\right)\rho \Delta t$. The total number of filaments that are crosslinked (i.e., a part of the elastic network) at time t=2Δt is $N_{c}\left(0\right)\exp\left[-\beta_{0;0}\Delta t-\beta_{\Delta t;0}\Delta t\right]+N_{b}\left(0\right)\rho \Delta t\exp\left[-\beta_{\Delta t;\Delta t}\Delta t\right]+N_{b}\left(\Delta t\right)\rho \Delta t$. By repeating this process, the total number of the crosslinked chains after *m* time intervals will become

(49) $$\begin{array}{c} N_{c}\left(m\Delta t\right)=N_{c}\left(0\right)\exp\left[-\sum_{i=0}^{m-1}\beta_{t=i\Delta t;0}\Delta t\right]+\sum_{j=0}^{m-1}N_{b}\left(j\Delta t\right)\rho \Delta te^{-\sum_{k=j+2}^{m}\beta \left(k\Delta t;\left[j+1\right]\Delta t\right)\Delta t}, \end{array}$$

which can be expressed in continuous form as

(50) $$\begin{array}{c} N_{c}\left(t\right)=N_{c}\left(0\right)e^{-\int_{0}^{t}\beta \left(t^{\prime };0\right)dt^{\prime }}+\int_{0}^{t}N_{b}\left(t^{\prime }\right)e^{-\int_{t^{\prime }}^{t}\beta \left(t^{\prime \prime };t^{\prime }\right)dt^{\prime \prime }}\rho \;dt^{\prime }, \end{array}$$

where the first term on the right hand side represents the number of the originally crosslinked chains surviving from the beginning, and the second term represents those chains that were crosslinked between t=0 and *t*, and still remain crosslinked [76]. For the first term, the elastic reference state remains the original undeformed configuration of the network at t=0. In contrast, the tension force that determines the rate $\beta (t_{1},t_{2})$ in the second term depends on the reference state at a time $t_{2}$ when the particular filament was re-crosslinked into the network at its force-free conformation.

As the reference states of the filaments are defined according to when they are crosslinked, the elastic energy density of the system is

(51) $$\begin{array}{ccc} F_{t.n}\left(t\right) & = & e^{-\int_{0}^{t}\beta \left(t^{\prime };0\right)dt^{\prime }}F_{p.n}\left(t;0\right)+\int_{0}^{t}\frac{N_{b}\left(t^{\prime }\right)}{N_{c}\left(0\right)}\rho e^{-\int_{t^{\prime }}^{t}\beta \left(t^{\prime \prime };t^{\prime }\right)dt^{\prime \prime }}F_{p.n}\left(t;t^{\prime }\right)\;dt^{\prime }, \end{array}$$

where the free energy form of the permanent network, $F_{p.n}$, can be chosen from the continuum models showed in the previous section, e.g., Equation (25). As with the rate of breaking, the expression for $F_{p.n}\left(t_{1};t_{2}\right)$ depends on the deformation at time $t_{1}$, measured with respect to the reference state (at $t_{2}$) when the fraction of the chains that is counted towards this contribution was re-crosslinked. The corresponding deformation tensor, measured at $t_{1}$ with respect to the reference state at $t_{2}$ is: $E\left(t_{1},t_{2}\right)=E\left(t_{1},0\right)E^{-1}\left(t_{2},0\right)$. With the formal expression for the dynamic free energy of the material, one can obtain the stress-strain relationship and other rheological characteristics of the transient network, once the regime of imposed deformation is specified. Two simple applications of the theory are explored below:

*Stress relaxation*. If the transient network is subjected to a constant uniaxial extension with stretch ratio *λ*, imposed instantaneously at t=0 (stretch ratios along the other two orthogonal directions are $1/\sqrt{\lambda }$ from the incompressibility condition), then the tensile stress along the stretching direction decays with time purely due to the breakage of crosslinks: any crosslinks that are re-established during this process remain in the force-free state and the second term in Equation (51) does not contribute to the response at all. The rate of this exponential decay depends on *λ* but remains constant over the course of relaxation:

(52) $$\begin{array}{c} \sigma_{\mathrm{ela}}=\sigma_{0}e^{-\beta t}, \end{array}$$

where $\sigma_{0}$ is the instant stress at t=0, which is equal to $dF_{p.n}/d\lambda$. The breakage rate of the crosslink in a primitive cubic cell of a 3-chain model, $\beta_{0}e^{fa/k_{B}T}$, can be calculated by taken the force in the equation as $\max[|f\left(\lambda x\right)|,|f\left(x/\sqrt{\lambda }\right)|]$, which is the maximum force acting on the three orthogonal chains of a crosslink. The explicit form of the force can be calculated by Equation (12). Assuming relatively stiff filaments with $c>c^{*}$, originally crosslinked near their force-free point (as most biological filaments studied in Figure 10 appear to be), the rate of relaxation takes the form

(53) $$\begin{array}{c} \beta \left(\lambda \right)\approx \frac{1}{\tau_{0}}e^{-E/k_{B}T}\cdot \exp\left[\frac{16\pi^{2}l_{p}}{5L}{\left(\lambda -1\right)}^{2}\right], \end{array}$$

where the additional exponential factor arising from the tensile force on a filament is approximated for small deformations (λ−1)≪1 to allow easy linearization. This characteristic exponential stress relaxation is a feature of all transient networks with a single value of the bare rate of crosslink breaking (a single value of the breaking barrier *E*). This is often the case in biological networks, as well as in the new vitrimer materials [77], while in physically crosslinked synthetic polymer networks one often finds a broad distribution of barriers, and the breaking rates. In such systems, the observed stress relaxation is usually the stretched exponential [76].

*Ramp deformation*. This deformation regime occurs when the material undergoes a continuously increasing uniaxial stretch, i.e., the stretch ratio changes with time as $\lambda =1+\dot{\gamma }t$, with a constant rate $\dot{\gamma }$. Contributions from the re-crosslinks can be usually ignored, since this rate remains constant while the rate of breaking continuously increases with time [78]. In this case the tensile engineering stress can be obtained as

(54) $$\begin{array}{ccc} \sigma & = & e^{-\int_{0}^{t}\beta \left(t^{\prime };0\right)dt^{\prime }}\frac{\partial F_{p.n}}{\partial \lambda }. \end{array}$$

**Figure 16:**
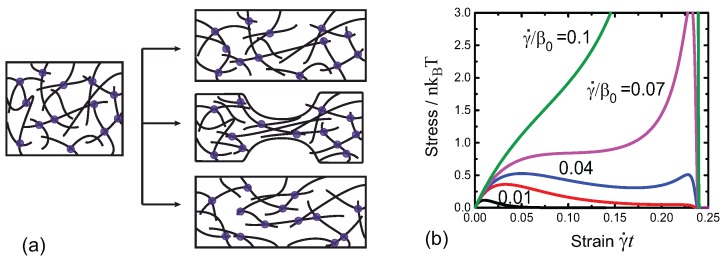
(**a**) Sketches of a transient filament network under an uniaxial stretch, behaving (i) elastically; (ii) showing the necking instability; (iii) plastically flowing; (**b**) Stress-strain relationship of a transient network under uniaxial stretch $\lambda =1+\dot{\gamma }t$ with different strain rates. At long times/high deformations, the highly non-linear response of filaments under tension leads to the total breakup of the network. Parameters used here are $a=10^{-3}L$ (bond characteristic length), c=0.4 (stiffness) and $x_{0}=0.8$ (pre-tension).

The 3-chain model is chosen for $F_{p.n}\left(\lambda \right)$ as an example. The network can respond in three distinct regimes: increase stress as an elastic solid, plastically flow as a fluid, or exhibit a necking instability when the two regimes coexist along the length of the sample, as illustrated in Figure 16a. The transitions between these regimes depend on how the imposed strain rate $\dot{\gamma }$ compares with the bare breaking rate $\beta_{0}$ (at zero tensile force on a crosslink). These regimes arise from the competition between the intrinsic non-linear elasticity of the material and the plasticity due to the breakage of the crosslinks.

Figure 16b illustrates a typical predicted response. When the reduced strain rate is large, the material just responses as pure elastic one as the chains have no time to break from the crosslinks. Note that the stress rapidly falls to *zero* when the strain approaches its critical value where the free energy of the network diverges, as the filaments experience infinitely large forces which break the filaments from the crosslinks. When the reduced strain rate is small, the stress of the material first increases due to elasticity of the network with its crosslinks still largely intact, and then reaches the yield point, following which the stress decreases (the network fluidises) due to an increasing number of broken crosslinks. When the reduced strain rate is moderate, the stress of the material first increases then decreases after a yield poin—however, the stress of the material will increase again due to a non-negligible number of the crosslinks surviving from the beginning and the increasingly high force some of these filaments exert. In this intermediate case the material shows the classical necking instability, where a strongly stretched region (plastic zone) and a weakly stretched region (elastic zone) coexist at the same level of stress across the sample.

## 5. Conclusions

This is a brief overview of the current state of theoretical understanding of physical properties of semiflexible filaments and their crosslinked networks, permanent or transient. This is a broad field with well over 30 years of active research and seminal contributions, and we could not possibly present all the references that perhaps ought to be here; our aim was to illustrate the key concepts as we see them presently, and hopefully assign these concepts to the right original work. In all cases we tried to leave the reader with the “final result”—a compact analytical expression for the relevant physical system, which can be manipulated and fitted to experimental data. The three main sections of this review start from the filaments themselves, examining their mechanical response (essentially force-extension, although the compression becomes a relevant matter for stiffer and athermal filaments). Here the main features are the non-linearity and divergence of the force-extension, the average natural extension of the stiffer filaments, and their metastable buckling on compression. The next section overviews the equilibrium elastic free energy of a randomly crosslinked network of such filaments, where the main physical effects are the specific strain non-linearity, the negative normal stress usually only encountered in auxetic materials, and the finite range of network stability. The final section examines the constitutive relation in a filament network with dynamic crosslinks, which is a very new topic with much less solid knowledge acquired to date. Still, we were able to demonstrate how the careful analysis of crosslink dynamics allows one to predict the network behavior in stress-relaxation and strain-ramp experiments.

## Figures and Tables

**Table 1:** Fitting parameters (c,x) for collagen, actin, vimentin, fibrin and neurofilament data obtained from experiment [29]. Also shown are the linear modulus $G_{0}$ extracted from the original data and used for scaling in Figure 8, the literature values of $l_{p}$ with their source, and the calculated mesh size $\xi =l_{p}\left(x/2c\right)$.

	$G_{0}(\mathrm{Pa})$	*c*	*x*	$l_{p}(\mu m)$	ξ(μm)
collagen	13.8	1.44	0.85	20.0 [42]	5.9
actin	95.2	1.36	0.85	17.7 [43]	5.5
vimentin	3.82	0.34	0.57	1.0 [44]	0.83
fibrin	18.9	0.25	0.40	0.50 [29]	0.40
neurofilament	2.83	0.14	0.15	0.45 [45]	0.24
